# Reconstruction Using Free Vascularized Fibular Grafts after Wide Resection of Humerus Chondrosarcoma in a Patient with Cleidocranial Dysplasia

**DOI:** 10.1155/2021/2302879

**Published:** 2021-03-08

**Authors:** Junya Shimizu, Kousuke Iba, Makoto Emori, Mikito Sasaki, Toshihiko Yamashita

**Affiliations:** Department of Orthopaedic Surgery, Sapporo Medical University School of Medicine, Sapporo, Japan

## Abstract

Cleidocranial dysplasia is characterized by hypoplasia of the clavicles, unerupted teeth, narrow pelvis, short stature, and craniofacial malformations. A cause of this skeletal dysplasia is heterozygous mutations of the runt-related transcription factor 2 gene (*Runx2*), a master regulator for bone and cartilage development. Chondrosarcoma is a primary malignant bone tumor that is usually treated by wide resection surgery. This report shows a case of a 25-year-old female patient with cleidocranial dysplasia who was affected with chondrosarcoma of the left humerus. We performed wide resection of the tumor and reconstruction of the large bone defect of the humerus using free vascularized fibular grafts. The patient preserved the hand function and activity of daily life as the same level as preoperative condition more than five years after the surgery.

## 1. Introduction

Cleidocranial dysplasia (CCD) is caused by heterozygous mutations of the runt-related protein 2 (*Runx2*) and is characterized by hypoplasia of the clavicles, unerupted teeth, narrow pelvis, short stature, and craniofacial malformations [1]. *Runx2* is a master regulator for bone and cartilage development and is essential for the commitment of pluripotent mesenchymal cells toward osteoblasts, as well as bone formation [2–4]. Previous studies have indicated that bone union could be delayed among patients with CCD [5, 6].

Chondrosarcomas are the second most frequent primary malignant bone tumors after osteosarcomas, which incidence is about one to two hundred cases per year in Japan, and are known to occur among patients over 50 years old [7]. Based on poor treatment outcomes for radiotherapy and chemotherapy, wide resection surgery is recommended as the only curative treatment. However, bony defects owing to the resection procedure must be appropriately reconstructed to preserve skeletal function. Several methods of surgical reconstruction after wide resection of a proximal humeral bone tumor include specialized endoprosthetic reconstruction of the large bone defect; recycled bone autograft using several procedures such as radiation, pasteurization, and liquid nitrogen freezing; vascularized fibula grafts (VFGs); and allografts [8–10]. Our previous study demonstrated favorable postoperative outcomes with free VFGs for surgical reconstruction of the proximal humeral defect after wide resection of a malignant tumor [11, 12].

We herein report the case of CCD affected with chondrosarcoma of the left humerus. We performed wide resection of the humerus with the tumor lesion and reconstruction of the large bone defect using free VFG, with more than 5-year follow-up after surgery.

## 2. Case Presentation

**Figure 1:**
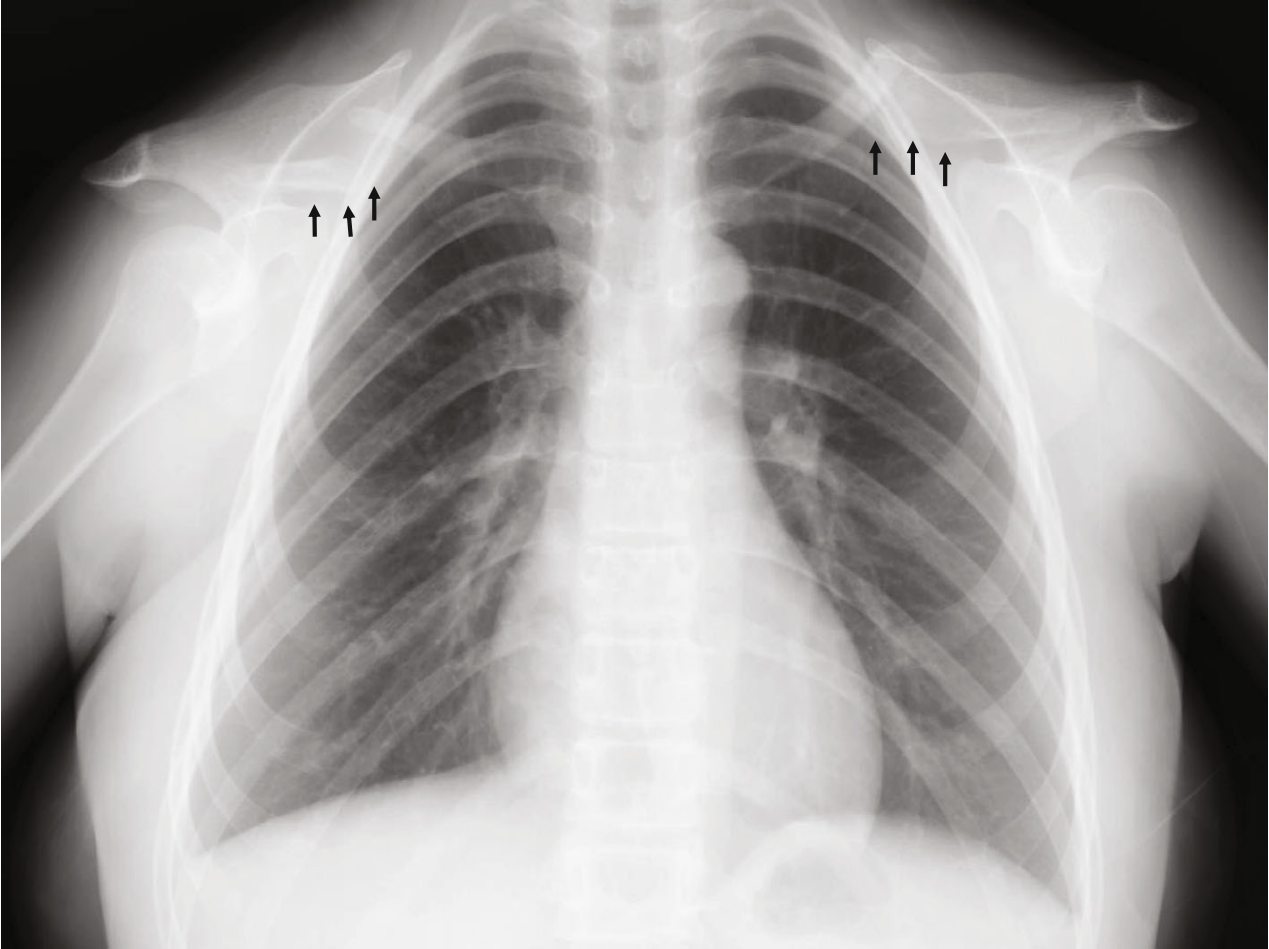
Plain chest radiograph plain chest radiograph (AP view) showing hypoplasia of the clavicles—a characteristic feature of cleidocranial dysplasia (arrows).

**Figure 2:**
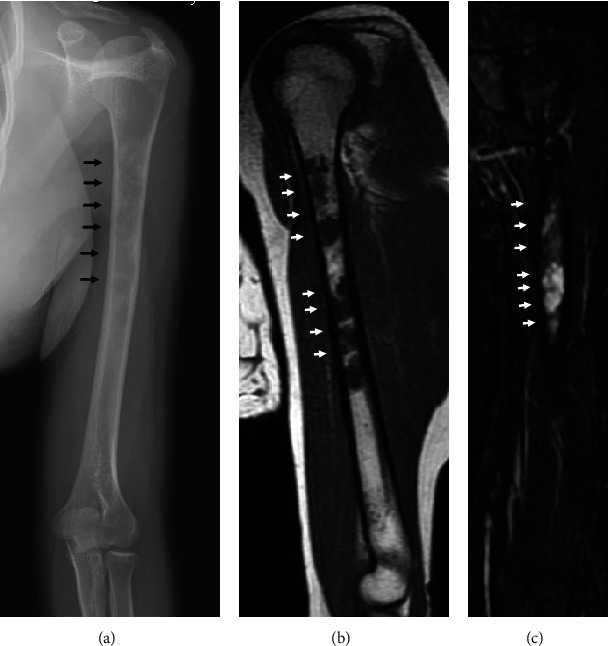
Plain radiograph and magnetic resonance imaging. (a) Plain radiograph showing radiolucent region and no periosteal reaction on the left proximal humerus (arrows). (b) Plain magnetic resonance imaging showing a low-signal-intensity region on T1-weighted scan. (c) High-signal-intensity lesion on short-tau inversion recovery.

**Figure 3:**
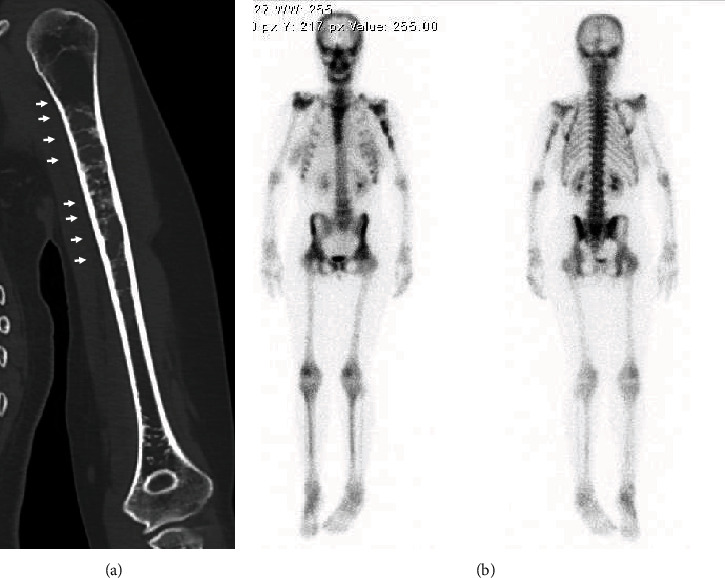
Plain computed tomography and bone scintigraphy. (a) Plain computed tomography showing calcification in the diaphysis of the left humerus (arrows). (b) Bone scintigraphy demonstrating increased focal uptake in the lesion (arrows).

**Figure 4:**
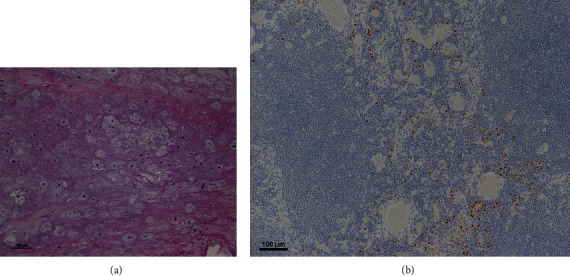
Histopathologic findings of the biopsy specimen. (a) Hematoxylin-eosin stain showing a pattern of chondrosarcoma (×100). This showed hypercellularity with prominent myxoid changes, which are characteristic of conventional chondrosarcoma. These were not specific pathological findings for CCD. (b) Immunostaining for *Runx2* (×100). This showed the cells were negative for *Runx2* antibody.

**Figure 5:**
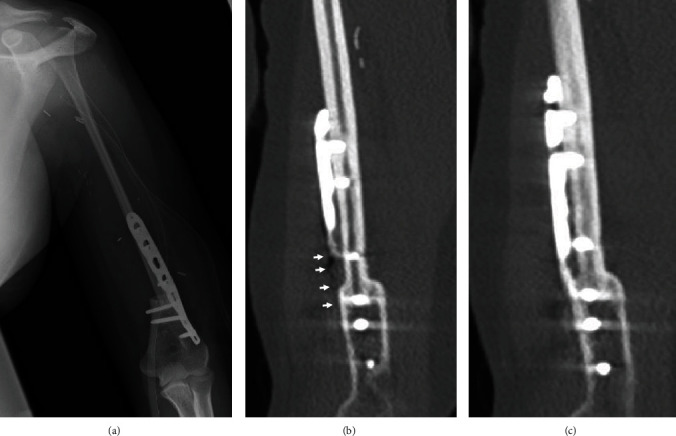
Postoperative findings of radiographs and computed tomography. (a) Postoperative radiographs showing the distal end of the free vascularized fibular graft fixed to the proximal stump of the humerus with a plate; sling procedure. (b) Postoperative CT scan showing the distal end of the grafted bone was fixed to the proximal stump of the humerus with a locking plate and suspended the head of the fibula for reconstruction of the shoulder joint with the tendons of the biceps femoris and palmaris longus. (c) Computed tomography taken five years postoperatively showing the formation of callus between the graft bone and humerus.

**Figure 6:**
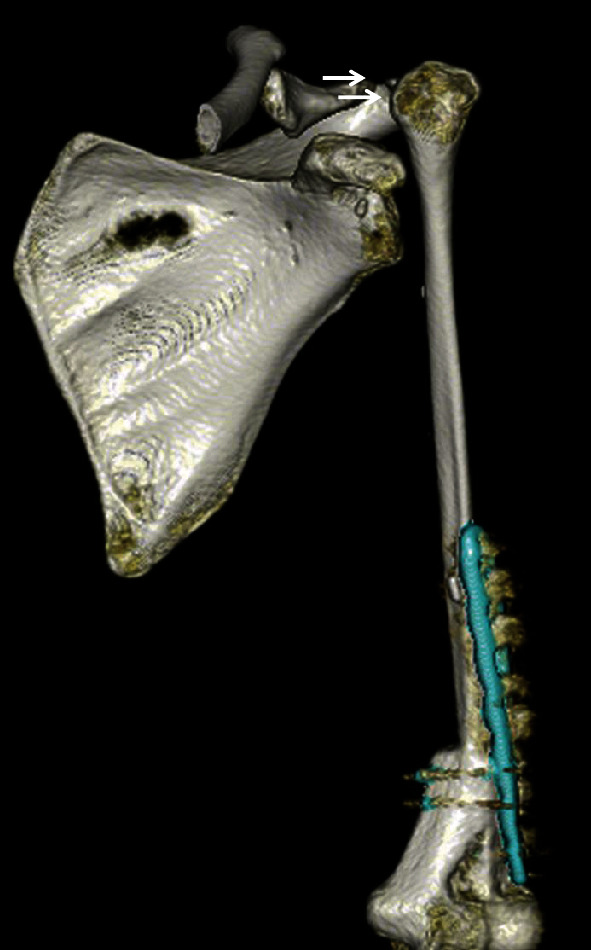
Postoperative 3D CT scan 3D CT scan showing the clavicle, scapula, humerus, and grafted fibula. Arrows indicates the location of the sling loop.

A 25-year-old female patient presented suffering from pain in the left upper arm over two months. The patient had been diagnosed with CCD at another hospital based on clinical features, radiographic findings, and genetic testing. The clinical characteristics included hypoplasia of the clavicles, narrow pelvis, short stature, and craniofacial malformations (Figure 1). Physical examination of the left upper arm revealed no findings of redness, swelling, or tenderness. Radiographic examination revealed radiolucent lesions without any periosteal reaction in the humerus (Figure 2(a)). Magnetic resonance imaging (MRI) showed a low-signal-intensity lesion on T1-weighted images and a high-signal-intensity lesion on short-tau inversion recovery (Figures 2(b) and 2(c)). In addition, a remarkable signal enhancement in the segment was found on contrast-enhanced T1-weighted MRI. A plain computed tomography (CT) showed calcification in the whole diaphysis of the left humerus (Figure 3(a)). Bone scintigraphy demonstrated increased focal uptake in the lesion (Figure 3(b)). For definitive diagnosis, we performed an open biopsy of the lesion. Based on histopathologic examination of the biopsy specimen, we diagnosed the patient with conventional chondrosarcoma in the left humerus (Figure 4(a)). On the other hand, we could not find any positive immunostaining cell for *Runx2* in the lesion (Figure 4(b)) and excluded metastasis of pulmonary or other lesion based on the findings of radiographs, CT, and bone scintigraphy. Thereafter, we performed wide resection of the lesion in the proximal humerus and sling procedure using free VFG for reconstruction of the large bone defect (Figure 5(a)). The distal end of the grafted bone was fixed to the proximal stump of the humerus with a locking plate. The tendons of the biceps femoris and palmaris longus, which were looped around the acromion with rotator cuff resection, were used to suspend the head of the fibula for reconstruction of the shoulder joint (Figures 5(b) and 6). The resected tumor was finally diagnosed as chondrosarcoma grade 2. Postoperatively, the upper extremity was fixed in an arm sling for 6 weeks. Three weeks after surgery, the patient began with a passive range of motion exercises of the shoulder and elbow. The patient was not administered radiation or adjunctive chemotherapy. Almost 2.5 years after surgery, the callus formation was found. Five years after the surgery, we confirmed the formation of callus between the distal end of the graft bone and the proximal end of the humerus (Figure 5(c)). Six years after surgery, we observed no recurrence, metastasis, or complications. The Musculoskeletal Tumor Society score, which includes assessment of range of motion, pain, stability, deformity, strength, function activity, and emotional acceptance, was 53%. In the latest evaluation, the patient maintained almost the same level of hand and elbow function and activity of daily life as that before the surgery and was satisfied with the postoperative outcomes.

## 3. Discussion

We report a rare case of chondrosarcoma of the left humerus in a 25-year-old patient with CCD. To our knowledge, this is the first report concerning the development of chondrosarcoma in a CCD patient, although there have been two case reports on CCD patients with other malignant tumors [13, 14]. Conventional chondrosarcoma mostly develops in individuals older than 50 years; however, our patient was a 25-year-old woman. We were unable to determine whether the pathophysiology and gene mutation in CCD contributed to the occurrence of chondrosarcoma at a younger age. However, further studies are warranted to elucidate the role of *Runx2* mutation in the development of malignant bone tumors. Several surgical procedures have been recommended for reconstruction of large bone defects after wide resection of malignant bone tumors, based on favorable postoperative outcomes [8–10]. We performed the sling procedure using free VFG after wide resection of a malignant humeral bone tumor in a younger patient. The sling procedure not only works as a functional spacer for the shoulder joint but also enables preservation of the passive scapulohumeral movement. Because it preserves glenohumeral joint function, satisfactory total functional scores have been reported after this procedure [11, 12]. While less than ideal, sufficient postoperative function for activities of daily life was maintained at a level similar to preoperative function in the present case.

We demonstrated favorable postoperative results including no recurrence or metastasis of the tumor and no complications in a CCD patient with chondrosarcoma. We believe that this case report could provide new clinical information. Previous studies using mouse models indicated that delayed bone repair was associated with heterozygous mutation of *Runx2*, which plays an important role in multiple processes of endochondral ossification [15]. However, it is uncertain whether patients with CCD constantly display nondelayed or delayed bony union after bone fracture or graft. Regarding the pathophysiology of the disease, we should consider the reduced potential for bone union in a patient with CCD for decision of surgical method. In our case, we performed the sling procedure using free VFG for reconstruction of the large bone defect. Generally, prosthetic reconstruction is preferable to bone grafting among patients with CCD because of an impairment of bone union. However, in this case, the remaining size of the humerus after wide bone resection was too small to be replaced by any available prosthesis, and young age of the patient could be considered a benefit to perform VFG. Houdek et al. [16] indicated that bony union was noted in 82% of cases by 2 years and in 97% of cases by 5 years after free VFG. Considering the period for grafted bone union after free VFG, 5 years in our case seems longer than that in previous reports. However, the complete bone union of the grafted fibula with the host humerus was finally obtained, and the patient was satisfied with the postoperative upper arm function. In this report, we showed the case of CCD affected with chondrosarcoma of the left humerus and believe a free VFG might be an option of surgical methods to reconstruct a large bone defect in skeletal dysplasia patient with an impairment of bone union.
